# Oral Water Has Cardiovascular Effects Up to 60 min in Shock Patients

**DOI:** 10.3389/fcvm.2021.803979

**Published:** 2021-12-20

**Authors:** Pierre-Grégoire Guinot, Maxime Nguyen, Valerian Duclos, Vivien Berthoud, Belaid Bouhemad, The water study group, Mohamed Radhouani

**Affiliations:** ^1^Anaesthesiology and Critical Care Department, Dijon Bourgogne University Hospital, Dijon, France; ^2^University of Burgundy Franche Comté, LNC UMR1231, Dijon, France

**Keywords:** oral water, shock, sepsis, cardiac failure, plasma volume, fluid therapies, acute circulatory failure, vasopressor

## Abstract

**Aim:** Little is known about the cardiovascular effects of oral water intake in shock patients. This study was designed to assess the effect of oral water on stroke volume and blood pressure during a 1-h time period.

**Method and Results:** This open-label, randomized clinical trial included patients admitted to intensive care with acute circulatory failure. Three ICU units at the anesthesia and critical care department of the Dijon Bourgogne University Hospital. Patients were randomized 1:1 to an intervention or standard care group. The intervention group received 500 ml of oral water while the standard care group received intravenous administration of 500 ml of physiological saline solution. Baseline SV did not differ between the two groups (36 ml [28;51] vs. 38 ml [30;51], *p* = 0.952). The number of patients who were fluid responders did not differ between the two groups [*n* = 19 (76%) vs. *n* = 18 (72%), *p* = 1]. The median change in stroke volume during the three time points did not differ between the two groups (*p* < 0.05). In the intervention group, blood pressure increased up to 60 min. In the control group, blood pressure quickly increased at the end of fluid expansion, then returned close to baseline value at 60 min.

**Conclusion:** Shock patients who were administered oral water experienced improvements in blood pressure and blood flow up to 60 min when compared with patients who received intravenous saline solution. Further studies are warranted to confirm these effects.

**Clinical Trial Registration:** www.clinicaltrials.gov, identifier: NCT03951519.

## Introduction

Volume expansion remains a daily challenge in critically ill patients (1). There is an abundance of published literature on the cardiovascular and metabolic effects of fluid therapies and their side effects in shock (2, 3). Fluid expansion has well-known cardiovascular effects, including increases in venous return, cardiac preload and cardiac output (CO), resulting in improved tissue perfusion (4). These effects are transient seeing as the increase in CO can be seen for a period of a few minutes (4). When administered repeatedly, these solutes lead to haemodilution, sodium overload, and renal failure (3, 5), which are associated with increased morbidity and mortality (2). Despite this abundant literature, to date there are no ideal fluid solution.

Before the development of the intravenous route, oral rehydration solutions were widely used. Oral rehydration was demonstrated to be associated with positive clinical effects such as an improvement in blood pressure, a lower fluid balance and a shorter hospital stay (6, 7). The absorption and homeostasis of water has been thoroughly studied in sports medicine and physiology (8). While the cardiovascular effect of volume expansion by saline has already been investigated in patients admitted to the intensive care unit (ICU), the cardiovascular effects of oral water is unknown. Only one before/after study have demonstrated an increase of stroke volume (9). Cardiologic studies have demonstrated that in non-shocked patients, oral water can change CO and blood pressure through various physiological effects: increase in blood volume, recruitment of splanchnic blood volume, activation of vasomotor tone, or change in heart rate (9–12). Oral water could be used to modulate orthostatic arterial hypotension. Oral water can modulate plasma volume and the response of the cardiovascular system to a stressful situation (13, 14).

Despite an abundant literature on oral feeding, we do not have data on the cardiovascular effects of oral intake of tap water in shock patients. Water is an easily obtained, natural product that can be administered orally, even in shock patients. Because we do not have data on the cardiovascular effects of oral water in shock patients, we designed a longitudinal randomized study over a 60 min period. This study aimed at describing the cardiovascular effects of oral water in patients with acute circulatory failure.

## Materials and Methods

### Trial Design

The study was approved by an Independent Ethics Committee (CPP 3 Nord-ouest CHU de Caen. 2018-A03368-47, NCT03951519). This study is a second part of an investigation aimed at describing the value of oral water in shock patients (9). All patients or their next of kin received written information about the study and gave their written consent to participate. This prospective, open-label, randomized, controlled, parallel-arm, monocentric clinical trial was conducted from May 2019 to February 2020 in three ICU units at the anesthesia and critical care department of the Dijon Bourgogne University Hospital (France).

### Participants

Inclusion criteria were participants who were aged 18 years or older, suffering from acute circulatory failure (systolic blood pressure < 90 mmHg, and/or mean blood pressure < 65 mmHg, and need for infusion of vasopressor amines, and/or skin mottling, and/or diuresis < 0.5 ml kg^−1^ h^−1^ for 2 h ≥, and/or arterial lactate level > 2 mmol/l), with stroke volume change following passive leg raising over 10% (preload dependency), and with no contraindication to a nasogastric tube. The exclusion criteria included permanent atrial fibrillation, hypothermia, concurrent participation in another study, and pregnant women.

### Intervention

The intervention consisted of oral tap water with a low mineral content (Cristaline™; Ca^2^+: 64 mg/l; Mg^2−^: 10 mg/l; Na^+^: 89 mg/l; K^+^: 3 mg/l; ${\text{HCO}}_{3}^{-}$:245 mg/l; ${\text{SO}}_{4}^{-}$:17 mg/l; Cl^−^: 140 mg/l) at room temperature (22°C). Over a period of 15 min, 500 ml of this water was administered to the patient through the nasogastric tube by using a pump. Standard care consisted of intravenous administration of 500 ml of physiologic saline solution (NaCl 9%) over a period of 15 min.

### Data Collection

All patients had been sedated by continuous infusion of propofol and opioid, and were fully adapted to volume-controlled mode with a tidal volume (Vt) set at 7-9 ml/kg of ideal body weight, and a positive end-expiratory pressure (PEEP) of 5-15 cmH_2_O. Ventilator settings, catecholamines, and sedation were not modified during the study period. The following clinical parameters were recorded: age, gender, weight, sedation, vasoactive agents, ventilation parameters, and main diagnosis. When a subject met the inclusion criteria, measurements of heart rate, blood pressure, respiratory variation of pulse pressure, echocardiography, and arterial/venous blood gas levels were obtained at each step of the study: baseline (time 1), immediately after administration (time 2), 30 min after administration (time 3), 60 min after administration (time 4).

Echocardiography was performed by a physician with echocardiographic certification (15). Tissue perfusion parameters were calculated from arterial/venous blood gas, as previously described (16) (Supplementary Material).

### Outcomes

The main outcome was the kinetic of stroke volume change between baseline (time 1) and 60 min after the end of fluid expansion (time 4). The secondary outcomes were the change of mean arterial blood pressure (delta MAP, %), the kinetics of arterial blood pressure, gap CO_2_, gap CO_2_/oxygen arteriovenous difference ratio, oxygen delivery (DO_2_), oxygen consumption (VO_2_), arterial lactate, hematocrit, sodium, and chloremia over a period of 60 min.

The plasma volume changes were evaluated by using the following formula: 100 ^*^ (Hct_pre_/Hct_post_ – 1)/(1 – Hct_pre_) at time 2, time 3, and time 4 (17).

### Randomization

Study participants were randomly assigned to one of two groups using a computer-generated randomization code (CleanWeb^®^ software). The randomization procedure was stratified by stroke volume change following passive leg raising with a 1:1 ratio. Although the research staff who collected data could not be blinded to group assignments, much attention was given to ensuring strict blinding during the data collection and echocardiography data analysis.

### Safety Assessment and Adverse Events

Endpoints and adverse events (vomiting, arrythmia, abdominal distention, worsening arterial lactate) were recorded by physicians affiliated with the hospital's clinical research division.

### Statistics

In absence of data, we performed a pilot study. The sample size calculation was based on volume expansion, where 500 ml of saline is usually associated with a mean SV change of 25% (+/−10%) for a mean baseline of 40 ± 10 ml (16). The inclusion of 25 patients can demonstrate a 10% difference in SV variation between the two groups with a power of 80% and a two-tailed *p-*value of 0.05. The normality of the data distribution was assessed using the Shapiro–Wilk test. Quantitative data were expressed as means ± standard deviation or medians [interquartile range], as appropriate, and qualitative data were expressed as numbers (percentages). Quantitative variables were analyzed by using bi-variate mixed linear modeling. The response variable was modeled depending on the group of randomization and the time of sampling (used as fixed effect) and the individual (used as random, intercept, effect). Normality of the random effect and of residuals distribution was checked graphically. When the fixed effect was significant for between group comparisons, a pairwise testing was carried out with Bonferroni *post hoc* correction. Statistical analysis was adjusted to baseline amount of fluid before randomization. Qualitative variables were analyzed by using Wilcoxon test or Chi-2 test. All hypothesis tests were two-sided, and the threshold for statistical significance was set at *P* < 0.05. All analyses were performed using R software (Version 1.1.447).

## Results

Fifty patients were included; there were no exclusions. The baseline characteristics were similar between the two groups (Table 1). Patients were admitted to ICU because of septic shock, cardiac arrest, cardiac failure, haemorrhagic shock, and following cardio-vascular surgery (Table 1). At baseline the mean amount of fluid did not differ between the two groups (20 ml Kg^−1^ [10;34] vs. 25 ml Kg^−1^ [12;41], *p* = 0.290). The number of patients with fluid responsiveness (i.e., SV increase of more than 15% between baseline and end of fluid expansion) did not differ between the two groups [*n* = 19 (76%) vs. *n* = 18 (72%), *p* = 1].

**Table 1:** Baseline characteristics of the study population.

**Variables**	**Standard group (*n* = 25)**	**Intervention group (*n* = 25)**
Age (years)	72 [62;77]	70 [62;76]
Male gender (*n*, %)	17 (68%)	14 (58%)
BMI (kg m^−2^)	27 [22;31]	29 [24;32]
SAPS 2	52 [47;62]	55 [49;61]
**Etiology of acute circulatory failure (** ***n*** **, %)**		
Post-operative (cardiac, vascular, thoracic)	4 (16%)	5 (20%)
Sepsis (pneumoniae, endocarditis, peritonitis, arthritis, cellulitis)	18 (72%)	17 (68%)
Cardiac arrest	1 (4%)	1 (4%)
Hemorrhage	2 (8%)	2 (8%)
**Vasoactive treatment (** ***n*** **, %)**		
Norepinephrine	25 (100%)	25 (100%)
Median dose	0.40 [0.17;0.81]	0.40 [0.17;0.83]
Dobutamine	6 (24%)	6 (24%)
Median dose (in patients treated)	7.5 [5.62;7.5]	10 [7.75;10]
Epinephrine	1 (4%)	0 (0%)
Baseline echocardiographic LVEF (%)	55 [50;65]	60 [48;65]
Stroke volume change with PLR (%)	14 [12;25]	14 [12;21]
Fluid balance before enrolling (ml kg^−1^)	20 [10;34]	25 [12;41]
**Respiratory parameter**		
Vt (ml Kg^−1^)	7 [6.8;7.4]	7 [6.8;7.4]
PEEP (cmH_2_O)	6 [5;8]	7 [6;10]

*BMI, body mass index; LVEF, left ventricular ejection fraction; PLR, passive leg raising; SAPS2, simplified acute physiologic score 2. Data were expressed as mean ± standard deviation, as median [interquartile range], or as numbers (percentage)*.

### Hemodynamic Changes Following Water Intake

**Figure 1:**
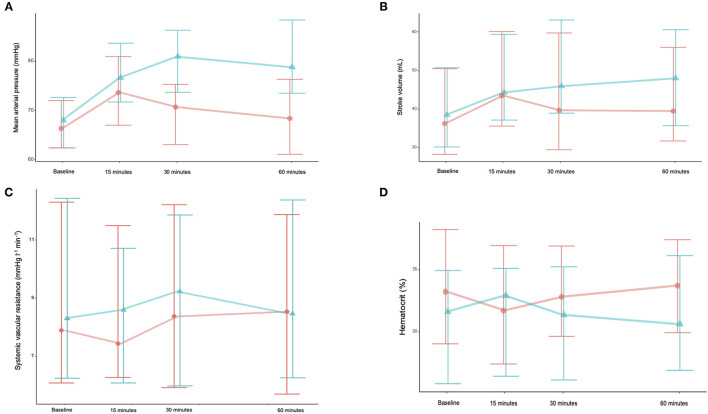
Changes over time in mean arterial pressure (MAP) **(A)**, stroke volume (SV) **(B)**, stroke systemic vascular resistance (SVR) **(C)**, and haematocrit **(D)**. Orange line is saline group, and green line is water group.

Baseline SV did not differ between the two groups (36 ml [28;51] vs. 38 ml [30;51], *p* = 0.952). The intervention and standard care groups exhibited significant differences in blood pressure over time (Table 2; Figure 1). In the intervention group, blood pressure, SV and CO increased up to the end of the study (time 4). In the control group, blood pressure, SV, CO quickly increased at the end of fluid expansion (time 2), then returned close to baseline value at 60 min (time 4).

**Table 2:** Change over time in the haemodynamic and tissue perfusion parameters.

		**Standard group (*n* = 25)**	**Intervention group (*n* = 25)**
Heart rate (BPM)	T1	94 [80; 107]	90 [80; 109]
	T2**	86 [77; 104]	89 [79; 104]
	T3**	90 [80; 102]	91 [71; 106]
	T4**	92 [80; 104]	91 [69; 104]
MAP (mmHg)	T1	6 [62; 72]	68 [62; 73]
	T2**	74 [67; 81]	77 [72; 84]
	T3**	71 [63; 75]	81 [74;86]*
	T4**	68 [61; 76]	79 [74; 88]*
SAP (mmHg)	T1	95 [83; 109]	99 [92; 110]
	T2**	110 [96; 126]	111 [105; 124]
	T3**	98 [89; 115]	114 [106; 125]*
	T4**	98 [88; 116]	117 [107; 124]*
Delta MAP (%)	NA	NA	NA
	T2**	6 [3; 19]	10 [2; 2]
	T3**	3 [−3; 10]	13 [4; 22]*
	T4**	1 [−4; 10]	14 [3;24]*
SV (ml)	T1	36.2 [28.2;50.5]	38.4 [30.2;50.7]
	T2**	43.5 [35.5;60.1]	44.3 [37.0;59.3]
	T3**	39.6 [29.4;59.7]	45.9 [38.9;63.1]
	T4**	39.4 [31.7;55.9]	48.0 [35.6;60.5]
Cardiac output (L min^−1^)	T1	3.40 [2.62;4.92]	3.89 [2.34;4.58]
	T2**	4.41 [3.47;5.13]	4.46 [2.55;5.28]
	T3**	3.56 [2.99;4.88]	4.25 [2.79;5.82]
	T4**	3.75 [3.12;5.07]	4.34 [2.93;5.13]
Arterial lactates (mmol l^−1^)	T1	3.80 [1.80;4.90]	3.30 [2.20;4.20]
	T2**	3.40 [1.40;5.00]	2.80 [2.10;4.20]
	T3**	3.40 [1.50;5.10]	2.60 [1.87;3.83]
	T4**	3.10 [1.50;5.60]	2.70 [1.90;3.60]
Haematocrit (%)	T1	33.2 [29.0;38.2]	31.6 [25.8;34.9]
	T2**	31.7 [27.4;36.9]	32.9 [26.4;35.1]
	T3	32.8 [29.6;36.9]	31.4 [26.1;35.2]
	T4	33.7 [29.9;37.4]	30 [26.9;36.1]

MAP, mean arterial pressure; SAP, systolic arterial pressure; SV, stroke volume; SVR, systemic vascular resistance. *Significantly different from baseline T1, Baseline; T2, immediately after intervention (15 min); T3, 30 min after baseline; T4, 60 min after baseline. *Significantly different between group by mixed linear modeling followed by pairwise comparison when needed. **Significantly different from baseline (regardless of the group) by mixed linear modeling.

### Secondary Endpoints

**Figure 2:**
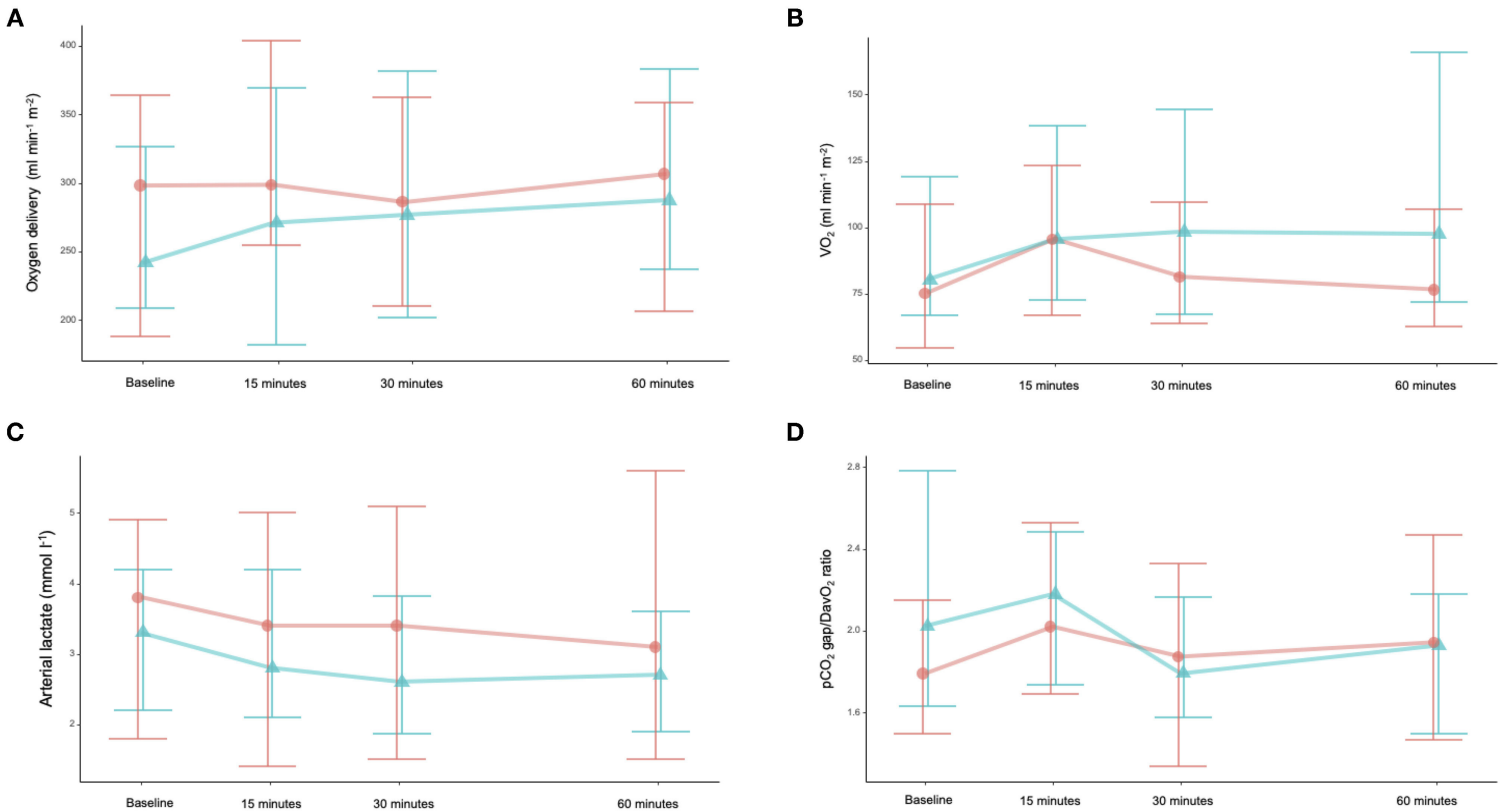
Changes over time in oxygen delivery (DO_2_) **(A)**, oxygen consumption (VO_2_) **(B)**, arterial lactate **(C)**, and gapCO_2_/DavO_2_ ratio **(D)**. Orange line is saline group, and green line is water group.

The evolution of oxygen delivery and oxygen consumption did not significantly differ between the two groups (Table 2, Supplementary Table 1 and Figure 2). They remained higher until the 60th minute in the intervention group. Arterial lactates decreased over time in both groups. The gapCO_2_ and gapCO_2_/DavO_2_ ratios did not change.

Oral water slightly increased hematocrit whereas saline infusion decreased hematocrit; both groups returned to baseline values at 60 min. After fluid administration, plasma volume changes differed between the two groups [8% (2-18%) vs. −2% (−5-4), *p* = 0.01). Then plasma volume decreased in the control group and increased in the intervention group [−4% (−6-0) vs. 2% (−2-4), *p* = 0.01].

### Safety Assessment and Adverse Events

The incidence of adverse events did not differ between the two groups (Supplementary Table 2). 18 patients (9 vs. 9, *p* > 0.99) died during their stay in the ICU. Natremia and chloremia did not changed.

## Discussion

The main result of the present study is that oral tap water increases CO and blood pressure up to 60 min. These effects do not differ in term of amplitude, but they are longer with administration of oral water than with the infusion of a saline solution.

These results may be explained by several known physiological mechanisms of oral water. The first and main effect is related to the pressor effect of tap water (10, 12, 18, 19). In the 2000's, Jordan et al. demonstrated that oral water has a pressor effect in patients with autonomic failure (20). Ingestion of water rapidly raises sympathetic activity, and norepinephrine plasma levels, which increases blood pressure (19–21). In the present study, blood pressure increased immediately after oral water administration because of the increase in CO and in systemic vascular resistance. The initial increase of CO may be due to the pressor effect of oral water. Hence, we noted hematocrit kinetics similar to what was previously observed with infusion of vasoactive agents, and plasma volume change was negative (17). Sympathetic activation may be due to several mechanisms such as distention of the abdominal viscera, osmolarity and temperature of the fluids (21–23). We choose to give tap water at room temperature because cold water has been demonstrated to be associated with lower pressor effects (22).

The second mechanism may be in relation to the absorption of water and its effect on plasma volume (24). Water was demonstrated to be quickly absorbed in the proximal part of small intestine. In healthy subjects, tap water is absorbed quickly, with a peak effect at around 15 min (24). On the contrary, we observed a delayed effect in our group of patients, with a peak effect 15-30 min after administration. The delayed gut absorption may be explained by shock-associated gut dysmotility (25). Despite this delay, we observed sustained hemodynamic effects 60 min after oral ingestion. In the same way, hematocrit kinetics inversely followed these of blood pressure.

Since water increases CO and blood pressure, we observed an increase in oxygen delivery. Oral water was associated with an increase in VO_2_ up to the final time point of the study, and this effect was not observed in the control group. It may be explained by several factors: DO_2_-VO_2_ dependency, the effect of oral water on gut oxygen balance, or body thermogenesis following water ingestion (26). It was previously demonstrated that feeding ICU burn patients is associated with a rise in gapCO_2_ that can be diminished by decreasing the amount of enteral nutrition (27). Oral water is able to increase thermogenesis through *b*-adrenergic stimulation, as observed with intravenous infusion of epinephrine (26). Increasing gut oxygen consumption could be a limitation of the hemodynamic effects of oral water.

### Clinical Implication

The present results demonstrated the effectiveness of oral water to improve blood flow and tissue perfusion. But these effects differ in term of duration from those observed with saline infusion. To date there are countless literature on fluid therapy, with a focus on renal effect and acid-base disturbance. No ideal fluid solution has been demonstrated. Interesting most of the literature has focused on fluid during shock resuscitation but maintenance fluid therapy and creep fluid may account for 30% of fluid balance (28). Oral water may not replace iv fluid but it's long-acting cardiovascular effects may be of interest during optimization and weaning process of shock patients supported by vasopressor and fluid (29). In addition, using oral tap water may be of interest in term of fluid balance because plasma volume effect and body elimination can be modulated by adding glucose or combining different type of water (tap, saline, sugar). According to Zdolsek et al. this approach can be associated with an increase of plasma volume up to 400 min with a limited volume (24).

Oral water may be harmful in specific subgroup such as acute heart failure, hemorrhage, or digestive ischemia. Few of the included patients were suffering of acute heart failure. Because the main underlying mechanism is the mobilization of the unstressed volume though pressor effect, giving oral water in hemorrhage shock may not be associated with fast hemodynamic effects. On contrary it could be associated with opposite effects (20, 30). Heart failure patients are very sensitive to preload and afterload status. Giving oral water during acute heart failure may be of caution because it can precipitate heart failure by increasing preload and afterload. Further studies evaluating different type of oral solute and their place in fluid therapy in ICU must be performed. These studies must focus on the type of oral solution (tap, saline, mixed…) and the group of ICU patients (sepsis, heart failure, hemorrhagic shock…).

We did not perform a double-blind study. This study must be considered as a preliminary study that provides data on cardiovascular effects of oral water. We assessed hemodynamic changes by using echocardiography and not cardiac output device. Echocardiography has already used in several studies to evaluate the hemodynamic effect of intravenous solutions with good accuracy (31, 32). We measured blood gas parameters from a central venous catheter and not from a pulmonary artery catheter. Since we performed repeated measurements of blood gas levels, mathematical coupling cannot be ruled out. But VO_2_ calculated from hemodynamic data is a valid alternative to VO_2_ derived from respiratory gas measurements.

In conclusion, the administration of oral tap water is associated with a sustained improvement in blood pressure when compared with patients who received intravenous saline solution. These results may be mainly explained by the vasopressor effects of oral water. Further studies are warranted to confirm these effects.

## Data Availability Statement

The raw data supporting the conclusions of this article will be made available by the authors, without undue reservation.

## Ethics Statement

The studies involving human participants were reviewed and approved by Comité de Protection des Personnes Nord-ouest 3, Centre hospitalier universitaire de Caen. The patients/participants provided their written informed consent to participate in this study.

## Water Study Group

Mohamed Radhouani, Tiberiu Constandache, Sandrine Grosjean, Pierre-Alain Bar, Pierre Voizeux, Emel Rafrafi, Audrey Martin.

## Author Contributions

P-GG, MN, and VB contributed to the conception and design. P-GG and MN searched the associated data and drafted the manuscript. BB provided the supervision support. MN performed data analysis. All authors contributed to the critical revisions and final approval of the manuscript.

## Conflict of Interest

The authors declare that the research was conducted in the absence of any commercial or financial relationships that could be construed as a potential conflict of interest.

## Publisher's Note

All claims expressed in this article are solely those of the authors and do not necessarily represent those of their affiliated organizations, or those of the publisher, the editors and the reviewers. Any product that may be evaluated in this article, or claim that may be made by its manufacturer, is not guaranteed or endorsed by the publisher.
