# Supplementary material for: Oral Water Has Cardiovascular Effects Up to 60 min in Shock Patients
Source: Front Cardiovasc Med. 2021 Dec 20;8:803979. doi: 10.3389/fcvm.2021.803979 (PMC8722716; doi:10.3389/fcvm.2021.803979)
Supplement: Supplementary file 1 [file Table_1.docx]

**Table 1. Change over time in the hemodynamic and tissue perfusion parameters and biological variables.** **^*^**: Significantly different from baseline T1: Baseline, T2: immediately after intervention (15 min), T3: 30 minutes after baseline, T4: 60 minutes after baseline. CVP: central venous pressure, DO_2_: oxygen delivery, VO_2_: oxygen consumption; ScVO_2_: central venous saturation. **: Significantly different from baseline (regardless of the group) by mixed linear modelling

|  | | **Standard Group**  **(n=25)** | **Intervention Group**  **(n=25)** |
| --- | --- | --- | --- |
| **CVP (mmHg)** | T1  T2**  T3**  T4 | 9 [6; 11]  11 [8; 15]  10 [8; 13]  10 [7; 12] | 10 [8; 12]  11 [9; 12]  12 [10; 13]  12 [9;14] |
| **ScVO_2_ (%)** | T1  T2  T3  T4 | 72.9 [60.5;77.8]  72.6 [63.6;81.3]  73.5 [61.8;78.1]  68.5 [63.4;77.0] | 67.8 [59.1;75.8]  64.0 [57.7;73.2]  64.5 [59.6;70.6]  63.6 [57.5;70.2] |
| **DO_2_ (ml min^-1^ m^-2^)** | T1  T2**  T3**  T4** | 298 [188;364]  299 [255;404]  286 [210;362]  307 [207;359] | 242 [209;327]  271 [181;369]  277 [202;381]  287 [237;383] |
| **VO_2_ (ml min^-1^ m^-2^)** | T1  T2**  T3**  T4** | 75.2 [54.5;109]  95.6 [66.7;123]  81.3 [63.9;109]  76.5 [62.6;107] | 80.2 [66.7;119]  95.4 [72.8;138]  98.3 [67.1;144]  97.4 [71.8;166] |
| **pCO_2_ gap (mmHg)** | T1  T2  T3  T4 | 8.00 [4.70;9.70]  8.00 [6.80;9.70]  6.90 [5.75;10.1]  8.20 [6.80;11.2] | 9.80 [6.33;11.0]  10.9 [7.85;14.1]  9.10 [7.15;11.2]  9.10 [6.80;11.9] |
| **pCO_2_ gap / DavO_2_ ratio (mmHg)** | T1  T2  T3  T4 | 1.79 [1.50;2.15]  2.02 [1.69;2.53]  1.87 [1.34;2.33]  1.94 [1.47;2.47] | 2.02 [1.63;2.78]  2.18 [1.74;2.49]  1.79 [1.57;2.17]  1.93 [1.50;2.18] |
| **Na+** | T1  T2  T3**  T4 | 138 [134;141]  138 [134;140]  136 [134;140]  137 [134;140] | 139 [137;141]  138 [135;141]  137 [134;141]  138 [135;141] |
| **Cl-** | T1  T2  T3  T4 | 109 [106;114]  110 [106;114]  110 [105;113]  110 [105;112] | 109 [107;110]  109 [108;110]  107 [106;109]  108 [106;109] |
