# Supplementary material for: Oral Water Has Cardiovascular Effects Up to 60 min in Shock Patients
Source: Front Cardiovasc Med. 2021 Dec 20;8:803979. doi: 10.3389/fcvm.2021.803979 (PMC8722716; doi:10.3389/fcvm.2021.803979)
Supplement: Supplementary file 2 [file Table_2.docx]

**Table 2. Adverse events.**

| **Variables** | **Standard Group**  **(n=25)** | **Intervention Group**  **(n=25)** | ***p*-value** |
| --- | --- | --- | --- |
| **Arrythmia** | 0 | 0 | > 0.99 |
| **Vomiting** | 0 | 1 (4%) | > 0.99 |
| **Abdominal distension** | 1 (4%) | 0 | > 0.99 |
| **Worsening arterial lactate** | 6 (24%) | 4 (16%) | 0.463 |
| **Death** | 9 (36%) | 9 (36%) | > 0.99 |
