# Supplementary material for: Oral Water Has Cardiovascular Effects Up to 60 min in Shock Patients
Source: Front Cardiovasc Med. 2021 Dec 20;8:803979. doi: 10.3389/fcvm.2021.803979 (PMC8722716; doi:10.3389/fcvm.2021.803979)
Supplement: Supplementary file 3 [file Data_Sheet_1.doc]

**Allocation**

**Analysis**

**Follow-Up**

**Enrollment**

Assessed for eligibility (n=110)

Excluded (n=60)

♦  Not meeting inclusion criteria (n=48)

♦  Declined to participate (n=2)

♦  Other reasons (n=10)

Analysed (n=25)
♦ Excluded from analysis (n=0)

Lost to follow-up (n=0)

Discontinued intervention (n=0)

Allocated to intervention (n=25)

♦ Received allocated intervention (n=25)

♦ Did not receive allocated intervention (n=0)

Lost to follow-up (n=0)

Discontinued intervention (n=0)

Allocated to intervention (n=25)

♦ Received allocated intervention (n=25)

♦ Did not receive allocated intervention (n=0)

Analysed (n=25)
♦ Excluded from analysis (n=0)

Randomized (n=50)
